# Drying Technologies for the Stability and Bioavailability of Biopharmaceuticals

**DOI:** 10.3390/pharmaceutics10030131

**Published:** 2018-08-17

**Authors:** Fakhrossadat Emami, Alireza Vatanara, Eun Ji Park, Dong Hee Na

**Affiliations:** 1 College of Pharmacy, Tehran University of Medical Sciences, Tehran 1417614411, Iran; f-emami@razi.tums.ac.ir; 2 College of Pharmacy, Chung-Ang University, Seoul 06974, Korea; 1978ej@naver.com

**Keywords:** biopharmaceuticals, drying technology, protein stability, bioavailability, pharmacokinetics

## Abstract

Solid dosage forms of biopharmaceuticals such as therapeutic proteins could provide enhanced bioavailability, improved storage stability, as well as expanded alternatives to parenteral administration. Although numerous drying methods have been used for preparing dried protein powders, choosing a suitable drying technique remains a challenge. In this review, the most frequent drying methods, such as freeze drying, spray drying, spray freeze drying, and supercritical fluid drying, for improving the stability and bioavailability of therapeutic proteins, are discussed. These technologies can prepare protein formulations for different applications as they produce particles with different sizes and morphologies. Proper drying methods are chosen, and the critical process parameters are optimized based on the proposed route of drug administration and the required pharmacokinetics. In an optimized drying procedure, the screening of formulations according to their protein properties is performed to prepare a stable protein formulation for various delivery systems, including pulmonary, nasal, and sustained-release applications.

## 1. Introduction

The intrinsic instability of protein molecules is currently the predominant challenge for biopharmaceutical scientists [1,2,3]. Because of their higher molecular weights and diversity of composition, therapeutic proteins have much more complicated structures than conventional chemical drugs [3,4,5]. Exposure to some environmental stresses, such as pH extremes, high temperatures, freezing, light, agitation, sheer stress, and organic solvents, can cause protein instability [4,5]. Since proteins can be degraded easily during manufacturing and storage, some strategies are suggested to improve protein stability, including the addition of stabilizers, protein modification with biocompatible molecules, nanomedicine, and nano- or micro-particle technology [6,7,8,9,10,11,12,13].

Drying strategies that process and dehydrate proteins to produce more stable protein formulations in the solid state are frequently used for biopharmaceuticals that are insufficiently stable in aqueous solutions [14,15,16]. Solid dosage forms of proteins are less prone to shear-related denaturation and precipitation during manufacturing and storage [1,15,17,18]. Because water molecules can induce mobilization of therapeutic proteins and other additives, liquid formulations of proteins are more susceptible to unfavorable physicochemical degradation. Consequently, water removal and embedding of proteins in a glassy matrix are good approaches for improved storage against physicochemical protein degradation [1,5,18].

Dried therapeutic protein powders have shown good storage stability at room temperature (≤25 °C), and dehydration is an easy and economical approach [19,20]. Dehydration is not only a drying procedure for improving protein shelf-life, but may also be used for engineering protein particles for various routes of administration. Dried biopharmaceutical powders have gained popularity as inhalation preparations for pulmonary, nasal, and sustained drug-delivery systems [21,22]. Numerous reviews of drying strategies have been published [23,24]. However, most of these reviews focus on small molecules, and reviews of using drying methods to improve stability or pharmacokinetic properties of therapeutic proteins are relatively few [25,26]. Because proteins are sensitive to environmental stresses, the techniques available for producing dried biopharmaceuticals are limited by factors such as production time, temperature, and various process-related stresses [26]. The features and drawbacks of each drying procedure should be considered for rational selection of a drying method to improve the stability of therapeutic proteins for different drug administration applications [27]. In this review, the drying techniques of biopharmaceuticals are discussed, with focus on the selection of appropriate drying methods for improving stability and desired pharmacokinetic properties of biopharmaceuticals. Stabilizers for protein formulations and applications of dried-powder formulations to local or systemic drug delivery are also highlighted.

## 2. Drying Techniques

Generally, drying involves three steps, which may be operated simultaneously. First, energy is transferred from an external source to water or dispersion medium in the product. The second step is phase transformation of the liquid phase to a vapor or solid phase. Finally, the transfer of vapor generated away from the pharmaceutical product occurs. The characteristics of dried particles can be effectively influenced by process parameters, such as temperature, pressure, relative humidity, and gas feed rate, besides characteristics of protein formulations, such as composition and type of excipients, concentration of solutes, viscosity, and type of solvent [28] (Table 1). Drying based on the mechanism of removing water can be classified into subgroups. Drying can be performed using an evaporation mechanism, such as vacuum dying or foam drying; evaporation and atomization pathways such as spray drying (SD); sublimation mechanisms such as freeze drying (FD) and spray freeze drying; and supercritical fluid drying methods using a precipitation mechanism [27]. The most common drying techniques, namely freeze drying, spray drying, spray freeze drying, and supercritical fluid drying will be discussed in this review.

### 2.1. Freeze Drying (FD)

The most common drying method for therapeutic proteins is FD [14,26,29], which has been used for many therapeutic proteins, including insulin dry powder for inhalation (Afrezza^®^, MannKind Corporation, Valencia, CA, USA) [14]. Since water molecules can induce mobilization of protein solution, protein stability can be improved by water removal and embedding of proteins in a glassy matrix through lyophilization [5]. FD is based on sublimation, where solid materials are directly transformed to the gaseous phase. The FD process involves the following three steps: freezing, primary drying, and secondary drying [1,5,18,30,31].

Freeze-dried proteins have greater storage stability than proteins in liquid dosage forms; however, this process applies freezing and dehydration stresses to the proteins, which may result in the alteration of protein structure [31,32,33]. Upon drying, the hydration shell surrounding the protein, which provides a protective effect, is removed. In addition, the protein solution becomes saturated because of ice crystal formation during the freezing process. The solute concentration, pH change, and ionic strength changes are formulation variables that should be considered for a stable protein formulation (Table 1) [1,33]. Recent infrared spectroscopic analyses have shown that acute freezing and dehydration stresses of lyophilization can induce protein unfolding [29,34]. To develop a successful protein formulation using an FD procedure, physical properties, such as glass transition temperature (T_g_) and residual moisture content, and operational parameters, such as pH and cooling rate, should be considered [29].

Furthermore, a hydrophilic molecule can be incorporated into the protein formulation as a lyoprotectant to overcome protein denaturation and preserve stability during lyophilization [1,17]. Stabilizers can protect proteins during freezing (cryoprotectants) and lyophilization (lyoprotectants) through water replacement and hydrogen bond formation (Table 2) [1,18,30,35]. Moreover, excipients have the potential to provide a glassy matrix to decrease protein-protein interactions and reduce protein mobility in a solid dosage form [36]. In summary, optimization of process variables and proper combinations of additives as stabilizers are requirements for stable freeze-dried products [35,36,37].

Liao et al. investigated the effect of excipients, such as glycerol, sucrose, trehalose, and dextran, on the stability of freeze-dried lysozymes using second derivative Fourier transform infrared (FTIR) spectroscopy [17]. They showed that the combination of trehalose and sucrose could raise the T_g_ of freeze-dried lysozymes, leading to the stabilization of lysozyme in freeze-dried formulations. This study indicated that the T_g_ of freeze-dried formulations and the protein stability during lyophilization were dependent on the excipient type and excipient to enzyme mass ratio. A recent study by Tonnis et al. [38] showed the influence of size and molecular flexibility of sugars on the stability of freeze-dried proteins, including insulin, hepatitis B surface antigen, lactate dehydrogenase, and β-galactosidase. Among freeze-dried proteins prepared in the presence or absence of disaccharide (trehalose) or oligosaccharide (inulin, 4 kDa; dextran, 6 kDa; dextran 70 kDa), those prepared in the presence of the smallest sugar (trehalose) showed high stability although trehalose-containing formulations had the lowest T_g_. In addition, the flexible oligosaccharide inulin was more stable than the rigid oligosaccharide dextran 6 kDa or 70 kDa. The combination of polysaccharide dextran 70 kDa and trehalose greatly increased the T_g_ of the formulation and improved the stability of proteins, as compared to formulations containing dextran alone. The flexible oligosaccharide inulin (4 kDa) provided better stabilization than the similarly sized but molecularly rigid oligosaccharide dextran 6 kDa. This study indicated that the combination of trehalose and dextran has an additive effect owing to the interaction potential of trehalose (water replacement) and enhanced T_g_ of dextran (glassy state).

### 2.2. Spray Drying (SD)

**Figure 1:**
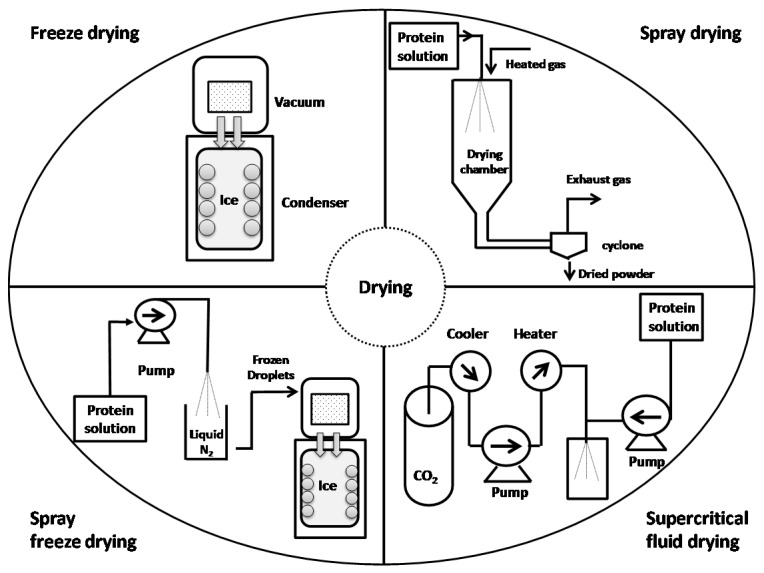
Schematic illustration of drying methods using freeze drying (FD), spray drying (SD), spray freeze drying (SFD), and supercritical fluid drying (SCFD) technologies.

SD is the most common particle engineering method that generates solid (particulate) proteins for pharmaceutical applications [41]. SD as a single-step process may provide dried protein particles with the required size and morphology [30,42]. SD technology comprises atomization, drying, and separation of particles. Protein solution is sprayed through nozzles into a drying chamber. Droplet formation and subsequent dehydration is performed very rapidly in a hot drying medium. The resulting protein powders are transferred into a cyclone (Figure 1). Owing to the short procedure time, SD is a mild technique for producing stable protein powders for inhalation and other applications [1,42,43,44,45].

Peclet number (Pe), which is the proportion of droplet evaporation rate and the diffusional motion of solutes in the SD method, can determine the morphology and density of the final particles that are either dense or hollow. The solute concentration, feeding flow rate, flow rate of hot air, solubility of additives through effects on evaporation rate, as well as inlet temperature are adjustable process parameters in the SD method. Large molecules with low diffusional coefficients having low solubility and high density, such as albumin and growth hormone, can prompt surface saturation at high temperatures. In addition, high surface-active agents such as leucine and trileucine would be located on particle surface and saturate the surface rapidly. Thus, for proteins and high surface-active agents, the solutes cannot diffuse to the center of droplets (Pe > 1) and generate hollow particles with low density that are suitable for pulmonary drug delivery [46]. Because of thermo-sensitivity of proteins, the loss of hydration layer in contact with hot air, as well as exposure to air/liquid interface at the droplet surface during atomization may cause degradation. Moreover, this engineering technique may induce shear stress in protein structure during the atomization step (Table 1) [1,2,5,44]. Excipients used as stabilizers may provide a functional shield surrounding the protein and prevent exposure of the protein to the interfacial surface or hot atmosphere [36]. The rational choice of stabilizers and optimization of the process variables are essential approaches to guarantee protein stability and achieve the desired particle properties.

Schule et al. studied the biophysical and conformational stability of spray-dried antibody-mannitol formulations [47] and concluded that antibody formulations during storage stability tests showed some levels of aggregates when measured by size-exclusion chromatography (SEC). FTIR analysis demonstrated that despite antibody aggregation during the SD process, the secondary structure of the antibody was conserved. Furthermore, their data suggested that the unfolding of protein structure was reversible and through reconstitution, the unfolded form of antibody formulation was refolded to a native form.

Ajmera et al. studied the SD of catalase formulations in the presence of some amino acids [1]. The ability of hydrophilic and hydrophobic amino acids to serve as protective agents during the SD of catalase was evaluated. The mean particle size of the spray-dried powder was estimated to be in the range of 3.3–4.8 µm, and amino acids have shown different stabilizing efficacies against the stresses induced by the SD technique. Spray-dried catalase, in comparison with the liquid form, showed greater stability. Arginine and histidine preserved the stability of enzyme formulations during SD. Due to its small size and surface activity, arginine exhibited fast diffusion to the droplet surface during droplet formation. Thus, arginine was localized at the droplet surface and protected the enzyme from air-liquid interface adsorption. Furthermore, hydrogen bond formation between amino acids and proteins in solid dosage forms plays a key role in the suppression of dehydration stress (water replacement). Charged hydrophilic amino acids, such as arginine and histidine, could form hydrogen bonds with catalase because of the presence of protonated nitrogen. X-ray diffraction (XRD) measurements have clearly shown that spray-dried pure catalase assumes a crystalline structure, while the enzyme remains in an amorphous state in the presence of histidine, and combination with arginine shifts its structure to a semi-amorphous state. A catalase-methionine formulation resulted in a crystalline product, which provided no protective effects. Despite its crystalline structure, glycine interacted with proteins as a water substitute and serves as a good stabilizer. Furthermore, a combination of arginine and glycine provides synergistic stabilizing effects and offers a potentially stable catalase powder preparation.

Li et al. prepared an inhalable protein formulation comprising 67% (*w*/*w*) sodium carboxymethylcellulose (Na-CMC) and 33% (*w*/*w*) alkaline phosphatase using SD [48]. They concluded that spray-dried alkaline phosphatase particles exhibited smooth surfaces. Particles with smooth surfaces are cohesive, which enhances the inter-particulate interactions and protein aggregation, causing low aerosol performance. Na-CMC as a polysaccharide in alkaline phosphatase formulations dried by SD may produce wrinkled protein particles. The roughness of protein surfaces may suppress unnecessary interactions and protein degradation. Spray-dried alkaline phosphatase-Na-CMC formulations maintained their stability immediately after preparation or following storage for three months, and subsequent protein powders produced in this manner have shown excellent aerodynamic performance.

### 2.3. Spray Freeze Drying (SFD)

Another effective and versatile technique for transforming protein solution into dried particles is SFD, which is the combination of traditional FD and SD processes. Atomization, fast freezing, and drying by ice sublimation are the three phases of SFD process (Figure 1). SFD is a method for preparing lyophilized protein powders with spherical microparticles [49].

Atmospheric freezing, spray freezing with compressed carbon dioxide, spray freezing into a vapor over a cryogenic liquid, and spray freezing into a liquid are different types of SFD methods [50,51,52]. SFD involves the atomization of protein solution via a nozzle at extremely low temperatures, and has potential applications for thermo-labile active pharmaceutical ingredients. Because of the critically low temperature, the atomized droplets are rapidly frozen. SFD methods may immobilize the protein and avoid exposure to air-water interface. The frozen micronized droplets are sublimated using a lyophilizer under vacuum to prepare a dried powder.

In SFD, the liquid solution is sprayed into a vapor via a nozzle using a cryogenic fluid, such as liquid nitrogen [43,44,53,54,55]. The chemical composition, protein solution concentrations, atomization rate, freezing rate, as well as the temperature of cryogenic liquid have key roles in determining the density and particle size distribution of spray freeze-dried powders [50]. Supercooling phenomena using a cryogenic liquid in SFD may reduce the undesirable effects of ice crystallization, pH shift values, as well as phase separation of the drug and excipient that have existence in FD. Moreover, since freezing is very rapid, there is not sufficient time for molecular rearrangements. Biopharmaceuticals are embedded amorphously in the excipients, minimizing the probability of protein crystallization and subsequent phase separation between the active pharmaceutical ingredient and stabilizers [56]. Furthermore, SFD can create such powders with the required density and particle size distributions for different pharmaceutical applications. Therefore, SFD is more advantageous than traditional FD [4,44,50].

Rogers et al. prepared spray freeze-dried insulin in the presence of tyloxapol and lactose as lyoprotectants [57]. Spray freeze-dried insulin in the presence of surfactant and sugar as lyoprotectants showed improved stability. The concentration of the covalent dimer of insulin in spray freeze-dried pure insulin preparations is only slightly higher than that of the unprocessed bulk insulin. Spray freeze-dried insulin with or without lyoprotectants showed little degradation, indicating that such preparations are as stable as the unprocessed native insulin. Maa et al. showed that SFD powders of influenza vaccines have superior stability compared with liquid formulations [58]. Processing other proteins such as rhDNase, anti-IgE monoclonal antibodies [21], calcitonin [53], and parathyroid hormone [44] using SFD provided stable dried powders with appropriate particle sizes and good flow properties.

Spray freeze-dried powders may be produced for different drug delivery system applications. Specific physical characteristics such as particle size distribution, density, surface area, and volume are required, depending on their application [50]. SFD typically produces highly porous particles with a high percentage of fine particle fraction (FPF) and proper aerodynamic behavior for pulmonary delivery [44]. In addition, spray freeze-dried particles have applications for needle-free intradermal injection system, nasal, colonic, and ophthalmic drug delivery, as well as in processing for microencapsulation platforms [50]. Spray freeze-dried particles with a geometric diameter of 7–42 µm and very low density, representing an aerodynamic diameter of 1–5 µm, could be effective in pulmonary drug delivery systems. However, for nasal delivery and intradermal injection systems, particles with geometric diameters of 25–70 µm and 34–50 µm are required, respectively [50].

### 2.4. Supercritical Fluid Drying (SCFD)

Supercritical fluid drying (SCFD) is an attractive alternative drying method, because dehydration can be rapidly accomplished in the absence of extreme temperatures. SCFD may produce large amounts of dried biopharmaceuticals with adjustable particle sizes and morphology [59]. SCF uses a material such as ethylene, methanol, or carbon dioxide above its critical temperature and pressure. The critical temperature of a liquid is the temperature at which its vapor cannot be liquefied, no matter how much pressure is applied. The pressure that is needed to condense a gas at its critical temperature defines its critical pressure. SCF exhibits the appropriate characteristics of gas and liquid, including penetration of gas and solubility of liquids. Density, viscosity, and diffusivity of SCF above its critical point are in the range of the gas and liquid states of the solvent. SCFD is a versatile process that can adjust the density of SCF and the solubility of a solute through modulation of pressures and temperatures used in the procedure.

Carbon dioxide is a non-toxic, non-inflammable, and relatively cheap fluid, with mild critical temperature (31 °C) and pressure (73 bar) for SCFD processes. Supercritical carbon dioxide (scCO_2_) may be used as a solvent or non-solvent in several SCFD applications, such as particle formation, chemical extraction, and purification. Rapid expansion of a supercritical solvent (RESS) and particles from a gas-saturated solution (PGSS) are two types of drying using scCO_2_ as a solvent. Gas anti-solvent (GAS), supercritical anti-solvent process (SAS), and solution-enhanced dispersion system (SEDS) processes are examples of anti-solvent scCO_2_ drying [15]. In previous studies, two theories explain the use of SCF for drying of protein products. In the first theory, SCFD is based on the anti-solvent and water extraction of SCF for protein formulations. Because of water extraction, the protein would be concentrated and precipitated. The concentrated protein solution is dried through the extraction of remaining water molecules using SFD. In the second theory, SCF is used as a propellant to enhance the atomization rate. In this process, SCF is dissolved at high pressure and the protein solution and SCF pass through a two-fluid nozzle, where the feed solution is atomized by the SCF, allowing a short drying procedure [60]. By monitoring the spraying gas flow rate, solution flow rate, solution concentration, nozzle size, temperature, pressure, and solvent, uniform spherical particles with distinct particle sizes and acceptable flow properties may be achieved [15,22].

### 2.5. Comparison of the Physical Characteristics of Dried Powders

**Figure 2:**
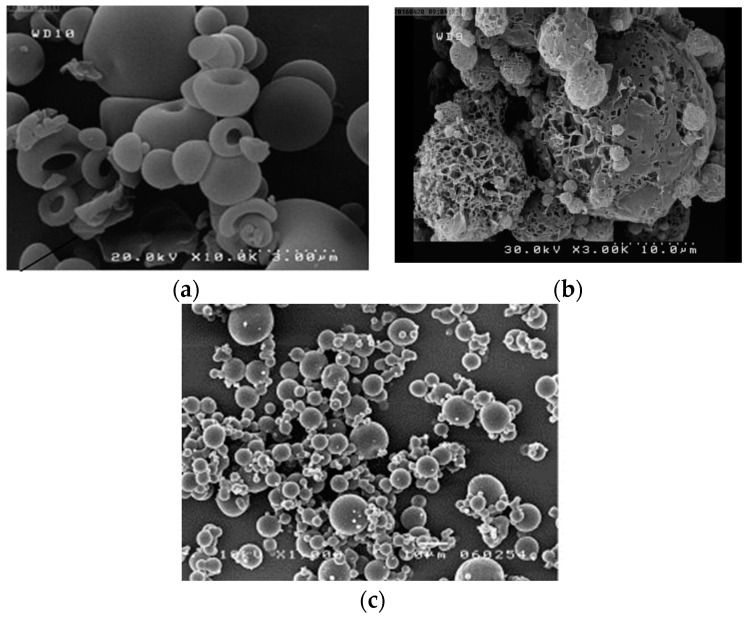
Scanning electron micrographs of dried IgG formulations produced using different drying methods: (**a**) SD [42], (**b**) SFD [52], and (**c**) SCFD methods [66].

As shown in Figure 2, immunoglobulin G (IgG) formulations prepared using different drying methods with trehalose as a stabilizer represent different particle morphologies. The diversity in the size and morphology of particles in SEM micrographs could be explained by the particle formation procedure used in different drying techniques [64,69].

The most common drying method for proteins and peptides is FD, which produces a dry cake. Intact cakes have very low residual moisture content with great long-term stability. FD powders of protein formulations after reconstitution can be used for parenteral drug delivery [33]. However, the use of FD for particle-based formulations, such as those for pulmonary drug delivery, is limited because it is not a direct particle formation method [37]. For particle formation, additional procedures to break the cake are necessary. Such additional processes may reduce the production yield and lead to poor control of critical particle properties, such as particle size, size distribution, density, and morphology [41].

SD is a well-established particle engineering process producing fine powders with smooth or shrunken surfaces. Generally, SD powders exhibit hollow particles with a dimpled shape. SD methods typically provide small particles between 2–10 µm and an FPF between 20–70%, respectively. Particles with low density have potential for use in pulmonary or nasal drug delivery applications [42,45]. SD processes can be used for microencapsulation of biopharmaceuticals as a controlled-release system [65]. Solid proteins prepared using the SFD method have specific morphological characteristics, namely presence of numerous pores on the surface and within the microparticles. Cross-sections of SFD particles have shown an internal porous structure similar to a honeycomb [59]. Compared with spray-dried particles, SFD particles showed higher sphericity and porosity, resulting in relatively low densities appropriate for inhalation [69]. When atomized with the same combination of feed solution, SFD produced a smaller aerodynamic diameter in comparison with SD particles because of their high porosity and relatively lower density. Small aerodynamic diameter means high FPF and good aerosol performance [50].

Saluja et al. used SD and SFD powders of influenza subunit vaccine with inulin as a stabilizer for inhalation [64]. SD techniques produced particles with a small median volume diameter (<5 µm), but SFD generated particles with a larger volume median diameter (>10 µm). Although these particles have different physical sizes, they have an acceptable aerodynamic size in the range of 1–5 µm for pulmonary drug delivery based on their bulk densities. SFD, rather than SD, is recommended for proteins sensitive to elevated temperatures [50].

Maa et al. reported that the same formulation of rhDNase and anti-IgE antibody prepared by SFD and SD resulted in different particle morphologies [21]. Differing morphologies observed in SEM images of the same protein formulation is indicative of particles with different sizes and densities. Although the diameter of SFD particles is approximately doubled (3.3 µm for SD vs. 7.7 µm for SFD), the FPF increased from 27% to 50%, which reflects an improved aerodynamic performance of SFD powder. These observations suggest that superior aerodynamic behavior by SFD particles is probably correlated with small aerodynamic size. Consequently, SFD powders, in comparison with SD powders, are reported to possess higher FPF and lower aerodynamic diameter, which result in higher aerosolization efficiency [52]. Because of rapid evaporation of water in the drying chamber, SD particles had smaller sizes resulting from particle shrinkage. With the SFD technique, immediately after atomization, droplets were frozen and solid ice crystals were produced throughout the frozen droplet. During lyophilization, the ice crystals sublimate, and particles with an interconnected porous structure are formed. SFD particles can maintain approximately 80% of their droplet size and therefore result in large-sized particles with high sphericity [50,64,69]. Low particle density can compensate for the large geometric diameter. However, particles with low density have lower mechanical strength and are more fragile, and are therefore more susceptible to degradation [70].

Unlike FD, the SFD method can adjust particle size, thereby producing better dispersibility. By managing morphological parameters, dense protein particles may be obtained for intradermal injection applications [58], and low-density particles can be used for nasal [19] and pulmonary drug delivery [44,53]. In addition, supercooling in SFD is advantageous for reducing the unfavorable effects of freezing-induced concentration of proteins that are present in FD. Furthermore, fast freezing during SFD may provide amorphous matrices that can trap proteins in a solid matrix and preserve protein stability [50].

Jovanovic et al. prepared dried IgG powder using an SCFD procedure, in which the product showed decreased aggregation at lower temperature [66]. They evaluated the stability of the antibody using trehalose and hydroxypropyl-β-cyclodextrin (HPβCD) as stabilizers. The occurrence of IgG aggregation in IgG formulations with lower pH values was also reported. Conversely, IgG-sugar formulations in the presence of ethanol as a co-solvent are less stable.

Consequently, all these techniques were useful for improving protein storage stability. These drying methods may be beneficial when the combinining of drying and particle engineering in one process is desired for preparation of dried protein powders.

## 3. Stabilizers for Dried-Powder Protein Formulations

Although proteins are formulated in a solid dosage form to maintain shelf-life stability, proteins are susceptible to various physicochemical degradations upon drying and in solid form [20]. According to the stress conditions encountered in each drying method, different degradation pathways may occur simultaneously. Various physical instabilities, such as unfolding, adsorption, denaturation, aggregation, and precipitation, may occur during the drying process. Deamination, oxidation, β-elimination, hydrolysis, racemization, isomerization, and disulfide exchange are irreversible reactions that are considered chemical degradations for biopharmaceuticals [71,72]. Physicochemical parameters such as moisture content, temperature, crystallinity of the formulation, and additives have a significant impact on these unfavorable reactions [20]. In addition, the protein sequence, hydrophobicity, isoelectric point (pI), and carbohydrate content play key roles in the susceptibility of proteins to inactivation. Degradation of protein products may result in reduced bioactivity with increased immunogenicity [20,73].

The stresses faced during drying procedures and in solid dosage forms remain a considerable challenge in biopharmaceutical development [18]. Rational selection of stabilizers may help preserve protein stability against stress-induced degradation [20]. The commonly used excipients are listed in five groups, namely proteins (BSA), amino acids (glycine, alanine), polyols (polyethylene glycol), carbohydrates (glucose, lactose, sucrose, trehalose), and others (surfactants, polymers, salts) [35], in Table 3. Cryoprotectants can preserve protein stability in solution and during freezing by preferential exclusion. Higher glass transition temperatures of protein formulations are required to provide stability to protein products. Moreover, lyoprotectants can provide thermodynamic stability of proteins during freeze-drying and dehydration process [17]. In instances where one stabilizer does not act as both a cryoprotectant and lyoprotectant, a combination of stabilizers may be beneficial to protect the proteins against FD-induced stresses [18]. As reported, protective agents can physically preserve protein stability via several mechanisms (Table 3). Among all stabilization pathways, water replacement and the glassy state hypothesis are more prevalent. Addition of excipients for stabilization may be required to stabilize proteins by either a thermodynamic stabilization pathway or by a kinetic mechanism [74].

### 3.1. The Water Replacement Hypothesis

Water plays a critical role in maintaining the native structure of proteins. During dehydration, water is removed and thermodynamic equilibrium between the native and unfolded state of the protein shifts to an unstable form. During drying, some stabilizers in the protein formulation can maintain the native protein conformation. These additives may enhance the free energy of protein unfolding reactions. Stabilizers with functional hydroxyl groups, such as carbohydrate and polyol, have the potential to form hydrogen bonds with bioactive proteins and substitute for the removed water molecules (anhydrobiosis). The hydrogen bonds formed between the carbohydrate and active protein are responsible for ameliorating dehydration stress in the solid state. As mentioned, this pathway can guarantee thermodynamic protein stability during the dying process [20,74].

### 3.2. The Glassy Matrix Hypothesis

The glassy matrix hypothesis is another mechanism that has a considerable effect on the kinetics of protein denaturation reactions. Because of molecular mobility, protein formulations are prone to chemical degradation, including protein aggregation and unfolding reactions. Some excipients, such as high molecular weight carbohydrates, may provide a rigid, glassy matrix that suppresses undesirable degradation reactions (glass dynamic hypothesis). Protein molecules can embed in a solid glassy matrix, which limits global mobility. Since protein mobility in a solid dosage form is restricted, possible protein-protein interactions are slowed, and the stability of proteins are preserved [20,74].

### 3.3. Reducing Surface Adsorption

Surfactants such as polysorbate 20 can localize at the interfacial surface owing to high surface activity. Thus fewer proteins are localized at particle surfaces and inhibit protein degradation [1].

## 4. Delivery and Pharmacokinetics of Dried-Powder Proteins

### 4.1. Pulmonary Delivery

The most predominant application of dried-powder protein formulations is for pulmonary delivery. In the pulmonary delivery of proteins, mucocilliary clearance, phagocytosis by macrophages, and alveolar proteolytic enzymes are critical biological barriers to local and systemic delivery. In systemic delivery, passage of the alveolar capillary membrane, proteases in blood circulation, renal clearance, and hepatic clearance are restricting factors that decrease the bioavailability of protein drugs [14].

#### 4.1.1. Local Delivery

Dried protein inhalation has been used as a topical drug delivery method for treating respiratory diseases. Direct drug delivery to the site of action provides high drug concentrations in the lung while reducing systemic blood circulation exposure to the drug, allowing safe therapies with high efficacies. The IL-4/IL-13 antagonist, IgG1, and an anti-IgE monoclonal antibody (Omalizumab^®^) have been administered as dried powder inhaler preparations for asthma, chronic obstructive pulmonary disease (COPD), or other related lung diseases [75]. For local administration, some important biological barriers should be considered to provide optimal therapeutic efficacy. Alveolar macrophage clearance of inhaled proteins is dependent on particle size, which can be decreased using both nanoparticles (diameter < 0.3 µm) and large particles (geometric diameter > 6 µm) [14]. Using SD method, insulin-loaded phospholipid/chitosan nanoparticles have been incorporated into microspheres in the presence of mannitol. Insulin-loaded microspheres have shown spherical morphology with suitable aerodynamic behavior as an inhalation preparation (aerodynamic diameter: 2–3 µm, density: 0.4–0.5 g/cm^3^) [76]. Edwards et al. showed that the in vivo sustained release of insulin in rats was achieved after inhalation of large porous particles (diameter > 5 µm, density < 0.4 g/cm^2^) prepared from poly(lactide-co-glycolide) (PLGA) [77]. Large porous insulin particles were inspired deep into the lungs and escaped pulmonary macrophage clearance mechanisms with a decreased immune response. Inhalation of larger porous insulin particles compared with smaller non-porous particles results in higher systemic bioavailability and suppression of systemic glucose levels for a longer period. This study showed that pulmonary protein delivery might provide highly efficient delivery of inhaled drugs into the systemic blood circulation. Additionally, large porous particles present better aerosolization efficiency. Higher aerosolization efficiency results in a decreased possibility of deposition losses before particle entry into the intrapulmonary airways, thereby enhancing the systemic concentration of an inhaled drug. Because of the relatively smaller surface areas of dried powders of large porous particles, less inter-particulate interaction occurs, resulting in decreased aggregation. Large porous particles based on a dipalmitoyl phosphatidylcholine (DPPC) combination as a dried powder inhaler preparation has revealed good characteristics for pulmonary delivery of peptides and proteins, such as parathyroid hormone, insulin, heparin, and human growth hormone. Moreover, in the presence of DPPC, the protein particles might have the potential for sustained release as an inhalant [14]. Among microparticles, hybrid large porous particles that have been prepared by the accumulation of nanoparticles using SD method are advantageous. The drug delivery and release patterns of nanoparticles combined with proper flow properties and aerosolization potential of large porous particles result in greater therapeutic effects of inhaled drugs [78].

Another clearance mechanism is the proteolytic enzymes present in the lung, which can degrade biomacromolecules by proteolysis. The addition of a protease inhibitor such as nafamostat, bacitracin, a trypsin inhibitor, chymostatin, leupeptin, bestatin, or aprotonin to the inhaled protein formulation may be useful for protecting proteins and leads to concentration enhancement [79,80]. When inhalable insulin was co-administered with nafamostat and bacitracin, its relative bioavailability increased, indicating that the proteins escaped from lung protease clearance [81,82].

#### 4.1.2. Systemic Delivery

Besides local inhalation therapies, pulmonary delivery of calcitonin, parathyroid hormone, and insulin (Exubera^®^ and Afrezza^®^) was developed for the treatment of osteoporosis, growth deficiency, and diabetes, respectively [71]. Systemic protein delivery through inhalation has been suggested to be a very promising alternative to parenteral delivery of proteins, and features the unique combination of a highly dispersed dosage form and an extended surface area to access the blood circulatory system [71]. Specific obstacles must be considered when a therapeutic protein is intended for systemic administration, because a sufficient proportion of drug molecule is required to be absorbed via the alveolar epithelium. For systemic administration of proteins, it is necessary to prevent degradation by blood clearance pathways, such as blood proteolytic enzymes, hepatic metabolism, and renal clearance to achieve an appropriate circulatory drug level leading to sufficient systemic bioavailability [14]. A major challenge to crossing the alveolar capillary membrane is the relative impermeability of the membrane to biomacromolecules. Macromolecule absorption through the double-layered phospholipid membrane by simple diffusion is limited by high molecular weight and hydrophilic nature of proteins. The absorption rates of macromolecules into the blood circulation are usually inversely related to molecular weight, with higher molecular weights leading to a higher T_max_ and lower C_max_ and partition coefficients [83]. There are some strategies to improve protein adsorption through the capillary membrane from the lungs into the blood. Absorption enhancers, such as surfactants, bile salts, cyclodextrins, citric acid, chitosan, and lipid-based carriers (liposomes), are suggested pathways to address the absorption issue [14].

### 4.2. Nasal Delivery

There is growing interest in the solid dosage forms of vaccines, which eliminate preservatives and the cold chain circulation for shipping and storage, while preserving protein stability at ambient temperature. Nasal delivery of vaccine formulations may be a good alternative to conventional vaccines [19]. Garmise et al. [84] demonstrated that an SFD powder of influenza virus increased local residence time and subsequently enhanced mucosal and systemic antibody production for nasal vaccination. Muco-adhesive compounds were characterized for their effects on the nasal residence time of vaccine powders in rats compared with in vitro data and stimulated immune responses. In vitro studies and in vivo imaging experiments revealed that sodium alginate and carboxy-methyl-cellulose powder combinations could enhance residence time in Brown Norway rats. It was concluded that nasal administration of dry powder influenza vaccine, especially in the presence of sodium alginate, could enhance serum and mucosal antibody responses. Furthermore, nasal delivery is an attractive vaccine platform for inoculating against other mucosal-transmitted diseases. Recently, Cho et al. [85] prepared a nasal powder formulation of salmon calcitonin (sCT) in the presence of a stabilizer (inulin or trehalose) and an absorption enhancer (chitosan, sodium taurocholate, or βCD) to improve its bioavailability. Dried powder inhalers of sCT were prepared by SD and novel SCF-assisted spray drying (SCF-SD). The in vivo absorption test in rats showed that spray-dried and SCF-spray-dried sCT powders increased the bioavailability of the peptide drug when compared with the nasal administration of unprocessed sCT, which was attributed to the absorption enhancer. Among the three absorption enhancers, chitosan-containing formulation showed the highest bioavailability, which was thought to be due to its properties of mucoadhesion or effects on tight junction to decrease mucociliary clearance. In addition, SCF-spray-dried sCT exhibited higher nasal absorption than spray-dried sCT in all formulations owing to the smaller size of particles. This study showed that SCF-SD method would be a promising approach for nasal delivery of dried powder inhaler of sCT.

### 4.3. Sustained-Release Delivery

Despite the high bioavailability of parenteral protein delivery, frequent injections result in poor patient compliance. Microsphere depot delivery system is designed to release the drug in a sustained manner over an extended period for systemic or local delivery. Spray freeze-dried BSA nanoparticles are encapsulated into PLGA microspheres to allow sustained release and to reduce the burst release pattern. The porous, solid protein particles break up into sub-micrometer particles followed by encapsulation into PLGA microspheres using anhydrous double emulsion techniques. In SFD, because of fast freezing and the absence of interfacial interactions, denaturation and aggregation of BSA were minimized, and the integrity and conformational stability of encapsulated BSA were preserved. The reduced burst release of BSA is attributed to the uniform distribution of protein nanoparticles in PLGA microspheres, which could be used as a sustainable delivery vehicle for biopharmaceuticals. Protein-PEG complexes are prepared by solubilizing BSA and recombinant human growth hormone (rhGH) in the methylene chloride phase. The organic phase containing PLGA and PEG/protein complexes was atomized through SD to prepare PLGA microparticles encapsulating proteins with good stability [86]. They exhibited sustained release profiles of BSA and rhGH and the released protein from the microsphere preserved their stability without aggregation. The protein microencapsulation method may prove to be a good platform for sustained delivery of therapeutic proteins that are not soluble in organic solvents. Cleland et al. [87] prepared PLGA microspheres containing recombinant human vascular endothelial growth factor (rhVEGF). The protein formulation was first sprayed into liquid nitrogen using an ultrasonic atomizer to produce rhVEGF PLGA microspheres. The SFD protein powder was added to a PLGA solution to prepare microspheres with a 9% *w*/*w* loading efficiency. SEC and mitogenic receptor-IgG binding affinity analyses showed that the stability and efficacy of rhVEGF were preserved through the SFD procedure. A study conducted by Al-Qadi et al. [88] presented a dried powder inhaler comprising microencapsulated insulin-loaded chitosan nanoparticles. The developed system was evaluated in rats to evaluate its potential for systemic delivery of insulin. The insulin-loaded chitosan nanoparticles were prepared by ionotropic gelation, followed by co-spray drying of nanoparticles with mannitol. Dried powder inhaler of insulin showed proper aerodynamic behavior for pulmonary delivery. The assessment of plasma glucose levels following intratracheal administration to rats showed that the microencapsulated insulin-loaded chitosan nanoparticles provided a sustained release of insulin with a more pronounced hypoglycemic effect than the insulin solution. This presents a good example of sustained release dried formulations for local or systemic delivery.

### 4.4. Enhancing Solubility and Bioavailability of Cyclosporine A

Cyclosporine A (CsA) is a cyclic undecapeptide drug used for the prevention of allograft rejection after lung transplantation [37]. As CsA has poor water-solubility, approaches to improve its solubility, bioavailability, and therapeutic efficacy have been designed. The solid dispersion of amorphous CsA in a methylcellulose-based matrix showed higher solubility than the physical mixture of the drug and methylcellulose. Additionally, an inhalable dry-emulsion preparation of CsA in the presence of glycerol monooleate as a surfactant showed better dissolution behavior and improved bioavailability compared with the native drug and its amorphous solid dispersion. The polymer-based amorphous solid dispersion of CsA as a dry powder inhaler revealed improved pharmacodynamic behavior as well as fewer side-effects [37]. Chiou et al. used a liquid impinging jet procedure to precipitate CsA nanoparticles with lecithin and lactose as stabilizing agents, followed by SD to produce nanoparticles for inhalation [89]. The aerosol performance of powders was evaluated using an Aeroliser^®^ dry powder inhaler with multi-stage liquid impinging jets. Nanomatrix powders exhibited appropriate flow properties (with a fine particle fraction of approximately 55%) and corresponding aerosol behavior. They demonstrated that the combination of precipitation with SD procedure has the potential to allow the design of protein particles with improved pharmacokinetics. Yamasaki et al. demonstrated that inhalable CsA nanoparticles in a mannitol-based matrix improved the dissolution of the drug [90]. CsA nanosuspensions were prepared using anti-solvent precipitation and subsequently, the micro aggregates were achieved using an SD procedure. The combination of amorphous CsA and crystalline mannitol showed similar drug content to the theoretical doses. The inclusion of mannitol as a wetting agent improved the dissolution rate of the drug, without significant impact on aerosol performance. This study indicated that the proper combination of drug and excipients could provide enhanced dissolution rate, with improved bioavailability of hydrophobic drugs, such as CsA.

### 4.5. Pharmacokinetics of Inhaled Insulins

In 2006, Exubera^®^ (Pfizer Inc., New York, NY, USA; Nektar Therapeutics, San Carlos, CA, USA) became the first inhaled insulin preparation approved by the US Food and Drug Administration (FDA) and European Medicines Evaluation Agency (EMEA). The Exubera^®^ device involves dispersion of insulin dry powder into aerosolized insulin within a large chamber, followed by patient inhalation. Exubera^®^ was delivered in a dry powder formulation containing amorphous insulin, glycine, mannitol, and sodium citrate as stabilizers. The dry powder of matrix particles was prepared using an SD method. The resulting powder, with low moisture content, showed good storage stability at room temperature for two years [91]. The onset of action was more rapid (32 min vs. 48 min, respectively) for Exubera^®^ than for subcutaneously injected (s.c.) insulin, and was comparable to lispro insulin. Furthermore, the duration of action of Exubera^®^ was longer than the duration of lispro (387 min vs. 313 min) and comparable to s.c. insulin [92]. However, after one year of commercialization, Exubera^®^ was withdrawn in 2007 because of its low bioavailability and enhanced treatment-related cost [92,93]. Some previous studies demonstrated the equivalence of Exubera^®^ to regular s.c. insulin in both type I and type II diabetes patients [94,95].

Another inhaled insulin product Afrezza^®^ (MannKind Corporation, Valencia, CA, USA) reached the market in 2014 and is currently the only inhaled insulin available in the market [91]. The formulation is based on Technosphere™ drug carrier technology, in which insulin is trapped and microencapsulated in small precipitated particles during self-assembly [96,97]. The precipitates are freeze-dried, but leaves small residual amounts of water [98]. Afrezza^®^ is a drug–device product containing a Technosphere insulin (TI) inhalation powder in single-use dose cartridges, which are administered with an inhaler. The particle size is 2–3 μm, with an internal porosity of approximately 70%, and once inhaled, it dissolves in the pH-neutral environment of the deep lung and rapidly releases insulin into the systemic blood circulation. TI inhalers mimic rapid-acting s.c. insulin analogs with a C_max_ of 15 min [91,98]. Pfützner and Forst compared the pharmacokinetics of 100 IU of Afrezza^®^, 10 IU with regular s.c. insulin, and 5 IU of i.v. insulin in healthy subjects [96]. Afrezza^®^ demonstrated very rapid absorption, with a mean T_max_ of 13 min vs. 121 min for regular s.c. insulin. In addition, Afrezza^®^ provided significantly higher bioavailability and faster absorption than Exubera^®^ (bioavailability relative to s.c. regular insulin of 26% and 10% respectively, and T_max_ of 13 min vs. 45 min). Afrezza^®^ still presents a much greater bioavailability than any other inhaled insulin formulations introduced to date. The observed pharmacokinetic profile could be explained by the highly efficient delivery of Afrezza^®^ particles to the deep lung (owing to the smaller particle size of Afrezza^®^). Because they are very small and porous, particles can be dissolved rapidly and provide high local concentrations of insulin that can be rapidly absorbed in the alveolae [98].

## 5. Future Perspectives and Conclusions

The development of therapeutic proteins has increased rapidly owing to their high therapeutic efficacy. Since the advent of human insulin in 1982, over hundreds of recombinant proteins have been developed and monoclonal antibody therapeutics market increased exponentially. Structurally modified proteins, such as PEGylated proteins and fusion proteins, and antibody-drug conjugate for cancer therapy have been recently available as therapeutics [99,100,101]. Biopharmaceuticals are available as liquid or solid dosage forms. In general, proteins have greater stability in the solid state than in the liquid state [33,41]. To achieve the desirable stability of proteins in formulations, solid-state dosage form is preferred to liquid-state dosage form, because it can provide a better shelf-life in storage. Numerous drying technologies, including FD, SD, SFD, and SCFD, have been used for dried protein formulations, and they have become an important method for the manufacture, storage, and delivery of protein-based biopharmaceuticals [14,25,26,37]. However, dried protein formulations still have challenges of stability and further optimization for desirable pharmacokinetic properties [3,83].

Currently, many dried protein powders have been produced through SD methods and other drying methods are still used only at the laboratory scale. In the future, drying methods at the laboratory scale must be scaled up for biopharmaceutical product development. Thus, modification of the existing drying technologies may be required. Achieving higher level of control of drying process parameters and performing under milder conditions may contribute to the improvement of stability and quality of biopharmaceuticals.

Dried powder inhaler preparations have mainly focused on local drug delivery systems for treating pulmonary diseases. However, these dosage forms also have a potential for systemic administration of therapeutic proteins, as demonstrated by the approved inhaled insulin products (Exubera^®^ and Afrezza^®^) [91]. Inhalation delivery of proteins can be a good choice for systemic delivery of protein drugs because pulmonary administration generally leads to higher bioavailability of protein drugs than other nonparenteral administrations, such as nasal or transdermal delivery [14]. Pulmonary route leads to rapid drug absorption owing to large surface area, low thickness of the epithelium, and rich blood supply, and avoids hepatic first-pass metabolism [83].

Since the development of pulmonary protein administration has shown significant progress, it can be predicted that soon the inhalation of proteins may become the first practical alternative to parenteral delivery. Clinical trials, as well as experimental studies have demonstrated that insulin as an inhaler possesses similar pharmacokinetic properties to that of s.c. injection of regular insulin, and even better properties than those observed with s.c. administration of rapid-acting insulin analogs. Substitution of injection with inhalation would be preferred by patients. However, there is no improvement with respective developments. If inhaled insulin becomes successful for diabetic therapy in terms of bioavailability and treatment cost, it will most probably create opportunities for the use of other biopharmaceuticals as inhalation preparations. Recently, other proteins and peptides have been studied for treating diabetes through the pulmonary route [100,102]. The major concerns regarding the long-term application of protein inhalers, such as insulin, are the development of insulin-antibodies and lung safety issues, which should be considered in future studies. Additionally, there are not sufficient studies considering the economic aspects of healthcare systems.

In conclusion, the successful solid dosage form of protein formulations in the presence of stabilizing agents could be achieved using several drying technologies. Cryoprotectants and lyoprotectants can maintain the stability and bioactivity of biopharmaceuticals. In each drying method, critical process parameters should first be optimized. Finally, screening of formulations based on protein properties is performed to determine the optimal stable protein/stabilizer combinations for a sustained-release, pulmonary, or nasal formulation.

## Figures and Tables

**Table 1:** Comparison of characteristics of different drying technologies. Table adopted from [15,21,23,24,39,40].

Drying Procedure	Process Parameters	Stress	Advantages	Limitations	Typical Powder Characteristics
Freeze drying	Solute concentration Cooling temperature Freezing rate Drying temperature Drying pressure	Crystallization pH changes Dehydration stress Ionic strength change Interfacial stress (ice-liquid) Ice crystal formation	Elevated temperature not required for drying Accurately dosed Controlled moisture content Short reconstitution time Appealing physical form Homogenous dispersion Good for materials sensitive to air or O_2_	No particle engineering Expensive set up Long processing time Complex process Maintenance cost Exposure to ice-water interface Few months for large objects	Intact cake High surface area Uniform color Consistency Elegant cake appearance High strength to prevent cracking, powdering, or collapse
Spray drying	Solute concentration Feed flow rate Hot air flow rate (Inlet and outlet) Additive solubility Inlet temperature	Thermal stress Atomization stress Mechanical stress Interfacial stress (air-liquid) Dehydration stress	Simple Convenient system Cost effectiveness One step (Short process time) Scalability Repeatability Particle engineering Good aerosolization	Yield (50–70%) Unsuitable for materials sensitive to air Non-aseptic	Fine powder Hollow particle Shrinkage Toughening Spherical, ellipsoid, toroid, or dimpled shape
Spray freeze drying	Solute concentration Feed flow rate Solid content	Atomization stress Interfacial stress (air-liquid) Freezing stress Interfacial stress (ice-liquid) Dehydration stress	Fast freezing Particle engineering High yield Excellent aerosolization Aseptic drying	Three steps (Time consuming) High cost Fragile particles Complex Inconvenient (require liquid N_2_)	Spherical, porous particle Light weight Smooth surface Very low density High surface area
Supercritical fluid drying	Solute concentration Feed flow rate Co-solvent flow rate SCF flow rate Temperature Pressure Nozzle size	Atomization stress Dehydration stress	Fast process Particle engineering Mild process condition (mild temperature) Aseptic drying Scalability	Exposure to organic solvent Special set-up High cost	Spherical Smooth surface

**Table 2:** Studies of solid protein formulations prepared by different drying methods in the presence of stabilizers.

Process	Proteins/Peptides	Stabilizers	Mechanism of Stabilization	Applications		References
				Stability Improvement	Drug Delivery	
Freeze drying	IgG Lysozyme BSA Anti-IgE antibody	Trehalose, Sucrose, PEG PEG, Glycerol, Sucrose, Trehalose, Dextran Glucose, Sucrose, Maltose, Trehalose, Maltotriose Histidine, Arginine, Glycine, Aspartic acid	Glassy state, Water replacement Water replacement Glassy state, Water replacement Glassy state	✓ ✓ ✓ ✓	_ _ _ _	[61,62] [17] [63] [18]
Spray drying	IgG Trastuzumab Anti-IgE Mab, rhDNase Catalase Influenza vaccine Alkaline phosphatase Erythropoietin	Trehalose, Sucrose, Leucine, Glycine, Lysine, Phenylalanine Trehalose, HPβCD, βCD Mannitol, Trehalose, Sucrose Arginine, Glycine, Histidine HEPES buffer, Phosphate buffer Sodium carboxy methylcellulose Dextran	Glassy state, Water replacement Glassy state, Water replacement Glassy state, Water replacement Water replacement, Inhibit interfacial adsorption Buffer Glassy state, Water replacement Glassy state, Water replacement	✓ ✓ _ ✓ _ ✓ _	Pulmonary _ Pulmonary _ Pulmonary Pulmonary Sustained release	[42,45,47] [2] [21] [1] [64] [48] [65]
Spray freeze drying	IgG BSA Anti-IgE Mab, rhDNase PTH Calcitonin Influenza vaccine Influenza vaccine Insulin Anthrax vaccine	Leucine, Phenylalanine, Glycine, Trehalose Ammonium sulfate, Mannitol, Trehalose Mannitol, Trehalose, Sucrose Trehalose, HPβCD, Leucine, Citric acid Trehalose, HPβCD, Maltose, Tween80 HEPES buffer, Phosphate buffer Dextran, Mannitol, Trehalose, Arginine Trehalose, Lactose Trehalose	Water replacement Reduction specific surface area Glassy state, Water replacement Water replacement, Inhibit interfacial adsorption Glassy state, Water replacement, Inhibit interfacial adsorption Buffer Glassy state, Water replacement - -	✓ ✓ _ ✓ ✓ _ ✓ ✓ ✓	_ Sustained release Pulmonary Pulmonary Pulmonary Pulmonary Epidermal Enhance solubility Nasal	[52] [36] [21] [44] [53] [64] [58] [57] [19]
Supercritical fluid drying	IgG Lysozyme Lysozyme, Myoglobin Insulin	Trehalose, HPβCD Trehalose, Sucrose Trehalose, Sucrose TMC, Dextran	- Glassy state, Water replacement Glassy state, Water replacement Carrier	✓ ✓ ✓ ✓	_ _ _ Pulmonary	[66] [22,59] [67] [68]

Immunoglobulin G (IgG), polyethylene glycol (PEG), hydroxypropyl β cyclodextrin (HPβCD), β cyclodextrin (βCD), bovine serum albumin (BSA), N-trimethyl chitosan (TMC), recombinant human DNase (rhDNase).

**Table 3:** Additives used as stabilizers during drying procedures.

Stabilizers		Stabilization		References
		Mechanism	Process	
Proteins	Human or Bovine serum albumin	Water replacement (Hydrogen bonding)		
Amino acids	Glycine, Alanine, Histidine, Leucine Phenylalanine, Arginine, Aspartic acid	Water replacement Bulking agent Buffering agent Prevent protein-protein interactions	Freezing Dehydration Thermal stress	[1,18,52,74]
Polyols	Polyethylene glycol, Mannitol, Sorbitol	Water replacement Glassy state Increase matrix density	Freezing Dehydration	[74]
Carbohydrate (reducing and non-reducing sugar)	Fructose, Glucose, Lactose, Maltose, Maltodextrin Trehalose, Sucrose, Inulin, Dextran	Water replacement Glassy state (reduce global protein mobility) Reduce local protein mobility Protein-sugar interactions	Freezing Dehydration Thermal stress	[20,52,74]
Buffer and Salt	HEPES buffer, Citrate buffer, Phosphate buffer saline, Ammonium sulfate	Buffering agent	Freezing	[64]
Surfactant	Polysorbate 20, 80, Oleic acid, Sodium glycolate	Prevent surface adsorption (Reduce interfacial stress) Prevent protein-protein interactions (Prevent intermolecular interactions) Slow dissolution rate	Shear stress Interfacial stress Freezing Reconstitution	[20,74]
Polymers and Polysaccharides	Cyclodextrin, Dextran, PLGA, Hydroxy propyl β-cyclodextrin, Na-Carboxy methylcellulose	Glassy state	Freezing	[20]
Metals	Zinc	Reduce surface area	Freezing Interfacial stress	[36]
